# The social construction of fibromyalgia as a health problem from the perspective of policies, professionals, and patients

**DOI:** 10.3402/gha.v9.31967

**Published:** 2016-12-19

**Authors:** Erica Briones-Vozmediano

**Affiliations:** 1 Department and Faculty of Nursing and Physiotherapy, University of Lleida, Lleida, Spain; 2 Public Health Research Group of the University of Alicante, University of Alicante, Alicante, Spain

**Keywords:** fibromyalgia, qualitative study, patients, health professionals, health policies, gender perspective

## Abstract

This article is a review of the PhD thesis written by Erica Briones-Vozmediano, entitled, ‘The social construction of fibromyalgia as a health problem from the perspective of policies, professionals, and patients’. The findings show that in Spain, the fact that fibromyalgia (FM) lacks recognition still remains: in policies, in the clinical and professional fields, and in the patients’ social circle. These three spheres have an influence on how this disease is constructed on a social level. International health policy has not yet taken steps to reflect the emergence of this recently diagnosed disease. The care for patients suffering from FM, who are mainly women, leads to frustration among the healthcare professionals and desperation among the patients themselves, as a resolutive treatment for the disease is not existing. Patients show resistance at assuming the sick role. They want to carry on undertaking their daily activities, both in the public sphere and in the private one. Roles involving the gendered division of labour were found to follow a rigid pattern, both prior to and subsequent to the disease, as the causes that led to frustration for men or women differ according to activities that are socially assigned to them. In practice, FM is conceived exclusively as a women’s health problem, which may result in a gender-biased patient healthcare attention. It is recommended that the implementation of specific policies for FM which could resolve this evident shortcoming should take place. To draw attention on a social level to certain illnesses considered to be attributed to women, such as FM, is of utmost importance, in order to allow the patients to be socially recognised as suffering a real and disabling disease.

## Introduction

The doctoral thesis entitled ‘The social construction of fibromyalgia as a health problem from the perspective of policies, professionals, and patients’ was a research project on the emergence and visibility of fibromyalgia (FM) in Spain, from the approach of the agents involved. It was focused on the social impact and uncertainty surrounding FM, a chronic disease that consolidates a number of symptoms and skeletal muscle issues which are little understood. The clinical characteristics most reported which define FM are generalised and chronic skeletal muscle pain, chronic fatigue, insomnia, and depression (1, 2). Other illnesses may co-exist together with these main features and a wide range of other symptoms whose existence and intensity vary from one patient to another. This unspecific symptomology places a high strain on medical and psychological resources. Furthermore, there are no objective tests available to obtain a diagnosis, consistent biomedical changes are not always present, and the disease’s aetiology is unknown. FM’s diagnostic criteria were defined in 1990 by the American College of Rheumatology and reviewed in 2010, and it was officially recognised as a disease by the World Health Organization in 1992 (1, 3).

Gender differences in FM have been mainly studied from a biomedical approach, in terms of exploring similarities or differences in the severity of symptoms between men and women, or hypothesising why the prevalence is much higher in women (with a men/women ratio of 1:20) (4). The need for the exploration of different social repercussions for women and men suffering from FM was the impetus which motivated the study, since there is a systematic lack of knowledge on how gender relations determine the significance that the disease has for the agents involved and to understand the subjectivity and the significance which is attributed to the disease from the perspective of those who are affected by it, mainly women, the professional personnel who care for them, and the social policy context which constructs the disease as a women’s health problem with the inherent implications. Other research gaps that were attempted to provide answers were to improve knowledge on the problems of people affected by FM from a public health perspective and gender sensitivity centred on individual needs and understand how health professionals describe this group of patients, as well as identifying preconceived socio-cultural ideas on the disease and its patients. The literature among gender differences in FM is still scarce, and this was one of the first studies exploring such sensitive topics (5).

### Conceptual framework

The gender perspective and the biopsychosocial model of health and illness were used as the conceptual framework in this doctoral thesis to describe the social construction as a health problem and the consequences of FM on the people affected by it. When the attempt to approach health issues is taken from a gendered perspective, one of a healthy/unhealthy biopsychosocial nature is implicit (6). From the biopsychosocial perspective, health is a process which is influenced by the biological factors as well as socioeconomic and cultural, psychosocial, psychological, subjective, and gendered ones (7).

In biopsychosocial theory, health/illness is an integral process whereby body, mind, and context are considered as inseparable. Therefore, sociocultural contexts and different lifestyle conditions between men and women are important factors, which also have an influence on people’s health (8). To give an example – the financial, productive, and social conditions as well as social class (job and level of education) affect people’s health in general (9) – the worst environment leads to the worst state of health, above all for women, who are the most vulnerable as far as social exclusion is concerned (10, 11).

Gender inequality has an impact on both men and women’s health (12). Being a man or woman can mean being subject to different psychosocial factors and power relations (13). The gender division of social duties in men and women leads to taking on certain roles, socially assigned based on sex in the biological sense, which then becomes gendered roles (14). Those roles traditionally undertaken by women generally imply lifestyles that can become detrimental to health (15). Family responsibilities, above all the double shift of work both outside and inside the home, have repercussions in women’s health, as they have a higher morbidity rate and are more at risk of having worse health than their male counterparts, and, as a consequence, resort to making more use of the health service in general (16, 17). Cultural models and stressful circumstances which depict women’s lives can have negative repercussions in the development of FM (18, 19). In turn, gender expectations are relevant in the case of chronic diseases, whereby gendered psychosocial components are potentially explicit in the way of feeling and becoming ill; that is to say that differences in gender exist as far as physical sensitivity is concerned (women are more alert, they are more attuned to physical symptoms, and they communicate them more) (20, 21).

## Aims

**Fig. 1:**
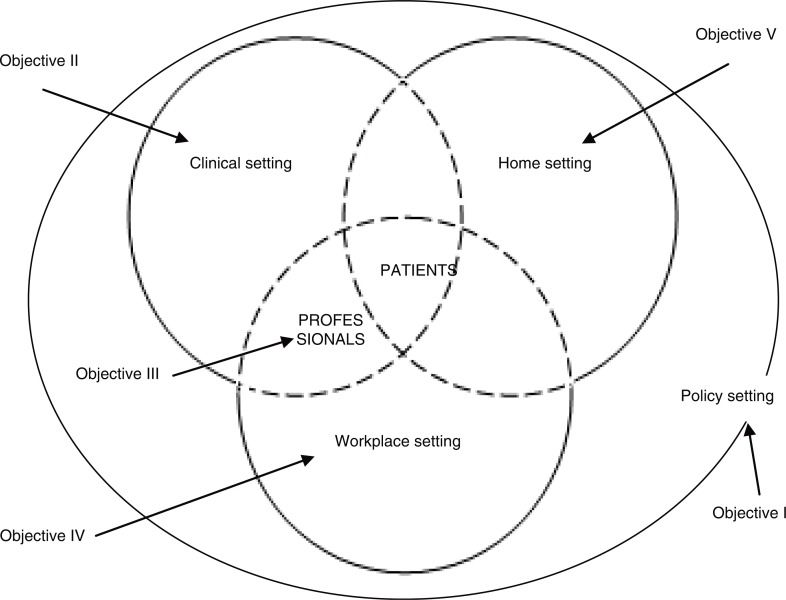
Specific objectives related to the settings involved in the social construction of fibromyalgia.

The main objective of this doctoral thesis was to explore the social construction of FM from the perspective of health policies, patients, and health professionals involved in their medical attention. Specific objectives studied were as follows: 1) the first specific objective was twofold: to examine as to what extent diseases which affect women such as FM were given priority under national health plans pertaining to the European Union and Latin America and regional health plans from Spain and to what extent these policies have produced specific clinical protocols for the management of FM, and to explore the visibility of FM in the European and Spanish parliamentary contexts. 2) To examine three clinical aspects of the management of FM: diagnostic approach, therapeutic approach, and the doctor–patient relationship, so as to probe into specific unsatisfactory areas lacking in the healthcare process, from two perspectives that differ from the disease itself: that of the health professionals’ perspective and that of the patients. 3) To explore health professionals’ discourses on patients afflicted with FM and the impact these discourses have on the quality of care patients receive. 4) To explore patients’ perceptions of the problems encountered in their working lives, in order to analyse how they deal with them and how they adapt to the limitations imposed on them as a direct result of the symptoms caused by this disease. 5) To describe how women diagnosed with FM suffer the impact on their private lives from a gender perspective. Figure 1 is a graph showing the organisation of social spheres in which each one of the independent objectives has been centred.

## Methodology

Each one of these individual studies that comprise this doctoral thesis has been dealt with from diverse methodological approaches and sources of information (Table 1).

**Table 1:** Summary of the methods applied in the doctoral thesis

	Part I	Part II	Part III
Study design	Policy review	Qualitative study	
Method	Systematic search and review	Personal interviews	
Participants	Health plans (international *n*=38, national *n*=17)	Professionals (*n*=12)	Patients (*n*=16)
	Spanish fibromyalgia protocols (*n*=6)		
	Parliamentary initiatives (*n*=65)		
Data sources	Secondary data	Primary data	
Analysis	Content analysis	Thematic analysis	
Setting	Policies	Clinical	Work/house
	International	National	
Year	2000–2012	2009	

### Part I: policy review

The methodology used in the first part of the thesis consisted of a systematic internet search and a systematic analysis on 1) the health plans in a national and international context, 2) the specific clinical protocols for FM in Spain, and 3) parliamentary initiatives in the European and Spanish context.

A systematic search was carried out on national health plans with strategic lines of action in the health section of official health institutions or health ministries of all countries pertaining to the European Union and Latin America. The plans selected were those that had unlimited internet access and which were available in English, Spanish, French, Portuguese, or Italian. Finally, 37 national health plans were analysed, the latest ones available at the time, to check if they included FM among the health problems for which included strategic objectives and lines of action. The same was done with the 17 regional health plans of Spain (in Spanish), recruited through visiting the webpages of the 17 autonomous regions of Spain. As well, at those webpages, specific protocols for the clinical management of FM were searched, but only 6 of the 17 autonomous regions already had one. Those protocols were read and analysed. The time frame covered was 2000–2010.

To review European and Spanish parliamentary initiatives, three different sources were consulted: the European parliament (22), the Spanish Congress of Deputies (23), and the Spanish Senate (24). In total, 65 parlamentary initiatives on FM were recruited and analysed. The first parlamentary initiative was produced in the year 2000 and the last one in 2012.

### Part II: qualitative study

This is a qualitative, explorative, and descriptive project, comprising a total of 28 personal interviews with 16 FM patients and 12 interviews with healthcare professionals.

In all the interviews performed, in compliance with the principles laid out by the Declaration of Helsinki and the Belmont Report, prior to the field work, ethical approval from the University of Alicante was obtained. Prior to data collection, an explanation was given to participants on the objective and methodology of the study, and they were offered the opportunity to question as far as any doubts were concerned. Confidentiality and anonymity were guaranteed, and informed consent was obtained from each participant (25).

#### Interviews with healthcare professionals

Twelve interviews with healthcare professionals were carried out. Twelve healthcare professionals from different fields implicit in the approach to dealing with FM (family medicine, rheumatology, occupational medicine, psychiatry, psychology, environmental medicine, and physiotherapy) from the Valencian Region in Spain were selected to take part in the study (Table 2). Nine were men, and three were women.

**Table 2:** Characteristics of health professionals interviewed (*n*=12) at the time of interviews

Interview code	Professional profile	Gender	Age	City	Institution	Role attending fibromyalgia patients
1	General practitioner	Female	42	Valencia	Healthcare centre	Follow-up, treatment
2	Rheumatologist	Male	35	Alicante	Hospital	Diagnosis, treatment
3	Rheumatologist	Male	44	Valencia	Hospital	Diagnosis, treatment
4	Physiotherapist	Male	53	Valencia	Hospital	Physical therapy
5	Occupational health physician	Male	45	Alicante	FM association	Disability assessment
6	Occupational health physician	Male	55	Valencia	Social security disability assessment team	Disability assessment
7	Occupational health physician	Male	42	Valencia	Social security disability assessment team	Disability assessment
8	Occupational health physician	Male	53	Valencia	Social security disability assessment team	Disability assessment
9	Psychologist	Female	39	Valencia	FM association	Psychotherapy
10	Psychiatrist	Female	43	Valencia	Hospital	Treatment
11	Psychologist	Female	42	Alicante	FM association	Psychotherapy
12	Behavioural specialist	Male	51	Alicante	FM association	Treatment

FM, fibromyalgia.

The interviews were carried out at professionals’ offices surgeries and lasted between 50 and 90 min, until information saturation was reached. Interviews were carried out on a semi-structured personal basis, based on an interview guide with open questions. The interview guide for professionals centred on professional competency on the speciality itself in dealing with FM patients, the experiences and associated problems relating to such patients, the procedural development, features of treatment procured, and perception of patient satisfaction with the procedures used, and the possibility of an improvement on approach.

The interviews were digitally recorded, transcribed literally, and imported to the Atlas.ti-7 software, with the help of which I carried out the analysis. To reach the specific objectives 2 and 3, qualitative content analysis (26) was the strategy used to analyse the interviews. The steps followed were: 1) phrases or paragraphs with the same meaning were identified from a repeated reading of transcriptions; 2) emergent codes were assigned to these units of meaning, following an inductive analysis model – these codes condense the meaning of the selected phrases and, thus, denote a further level of conceptual abstraction; and 3) the codes were ordered and grouped together forming themes or categories, which captured the perceptions of the professionals interviewed on the aspects on which each study was centred. The quotes appearing in each article were chosen on a basis of clarity and representation.

As a last step of the analysis of the interviews with health professionals, using the methodology of situational analysis (27), I constructed a situational map to graphically explain the variety of ideal professionals found. To establish some ideal types of professionals, a theoretical construct grounded in the empirical data which metaphorically captures their attitudes towards FM and its patients (28), a map was construed from the combination of two criteria: an ideal type was situated into one specific point of the graph, closer or farer to one of these criteria. Situational analysis, a qualitative methodology which has its roots in grounded theory, is useful to order the social complexity reflected in the discourses through graphically positioning the concepts implicated; in this case, this method was applied as a further analysis of the ideas that emerged in the previous analysis.

#### Interviews with persons suffering from FM

Sixteen interviews with 13 women and 3 men diagnosed with FM aged 24–61 were carried out (Table 3). Mainly women aged over 40 were selected, as these are the groups whereby the disease is most prevalent (29). Following a theoretical sampling technique, women were selected to represent various ages, family status, and occupational backgrounds. Men were selected purposively according to their capacity to contribute to the research question, since it was more difficult to recruit them. All participants were in paid employment when diagnosis was made; however, when the interview was carried out, the majority (n=10) were unemployed due to the progression of the disease. All participants were Spanish, with the exception of a man who was Ecuadorian. They all lived in the Valencian Autonomic Region (municipalities and towns from Alicante and Valencia) and had been diagnosed with FM by a Rheumatologist from the National Health System, which was used as a criterion for inclusion in the study.

**Table 3:** Characteristics of people affected by fibromyalgia (*n*=16) at time of interviews

Interview code	Age	Profession^a^	Marital status	Children
Woman 1	61	Inactive (*orange packing factory worker*)	Married	1 boy, 1 girl
Woman 2	52	Unfit for work	Divorced	1 boy
Woman 3	53	Inactive (*waitress in a family-owned bar*)	Married	2 boys, 1 girl
Woman 4	33	Inactive (*cook*)	Married	1 boy
Woman 5	52	Cleaner	Married	1 boy
Woman 6	44	Administrative assistant	Married	2 girls
Woman 7	38	Hairdresser	Lives with partner	No
Woman 8	29	Call centre operator	Lives with partner	No
Woman 9	45	Primary school teacher	Married	2 boys
Woman 10	59	Unfit for work (*hospital orderly*)	Married	2 boys
Woman 11	55	Unemployed (*shoe factory worker*)	Married	2 boys
Woman 12	51	Inactive (*shoe factory worker*)	Married	1 boy, 2 girls
Woman 13	24	Inactive (*waitress in a restaurant*)	Lives with partner	No
Man 1	42	Labourer	Divorced	2 boys
Man 2	54	Sick leave (*teacher*)	Married	1 boy
Man 3	48	Inactive (*truck driver*)	Married	1 boy, 1 girl

a In case of current inactivity, the former profession is indicated in italics.

The interviews were carried out in patients’ own homes. The approach used in the FM sufferers’ interview was that of relative subjects in their personal clinical history up to obtaining a diagnosis, the limitations and factors that make the symptoms worse, their experiences in their working lives, in the home, in interaction with the public sphere (institutions, professionals, and social network), and suggestions on ways to improve their quality of life.

To reach the specific objectives 2, 4, and 5, qualitative content analysis was also the strategy used to analyse the interviews with patients: reading the interviews, identifying meaning units, open-coding these sentences or paragraphs, and grouping the codes to form categories (26). The methodology of situational analysis (27) was also applied to establish ideal patient profiles as a last step of the analysis of the interviews with patients, following the same steps to order the emerging concepts from the previous analysis.

## Results

The main results of this PhD thesis are the following.

### Part I: from the policy perspective

#### Health policies: invisibility of FM and other ‘women’s’ diseases

Diseases that show a high predominance of women being affected, as is the case with FM, are not given priority in National and International Health Care Plans. Women’s healthcare tends to be linked to their reproductive organs, and the issue of gender is hardly noticeable.

National Health Plans of the international context (Europe and Latin America) do not include the approach to FM among the health issues, which are prioritised. That is to say, among the strategic lines of action, there are no interventions programmed to take on FM. This is also the case for other emergent issues which mainly affect women, which frequently co-exist with FM, such as chronic fatigue and chemical multiple sensitivity. The international health plan analysis shows that women’s health directly relates to that of a reproductive stance and does not consider other aspects of morbidity, which are more prevalent in women in comparison with men, or the inequality found in access to health care. Proof of this point is that the majority of said plans have specific sections on women’s health, relating to reproductive health or cancers linked to their sex.

In Spain, only 4 of the 17 regional health plans include the necessity of a chronic pain procedural approach for FM and chronic fatigue. Some of these regional health plans outline the necessity of developing procedures to deal with FM and chronic fatigue syndrome in primary health care. Presently, there are only six healthcare protocols on FM among the 17 autonomous regions, which is significant as to the lack of an official domestic strategy with regard to the disease. These clinical guidelines are meant to help healthcare professionals reach a diagnosis, by achieving this quickly and accurately – being aware of the difficulty of said diagnosis – and in pain management treatment, along with the improvement of patients’ quality of life. Even so, a gendered perspective is not rendered to protocols laid down by the different autonomous regions, and both consensus and unity are non-existent.

#### Parliamentary initiatives on FM: denounce of the invisibility of predominantly female diseases

Parliamentary initiatives, European ones as well as Spanish ones, reveal the lack of social recognition of the disease, its difficult diagnosis, the lack of resources assigned to the research and subsequent treatment, and the lack of recognition of the physical limitations involved affecting ability both in the labour market and in the home. In turn, the following points are outlined as being issues to be resolved: the improvement of patient health care, and an increase of effort and resources as far as research on the disease is concerned (expedite studies on its social, legal, labour, and political agenda repercussions).

The problem of FM has been introduced into European parliamentary debates stemming from the debate in 2005 on Gender Discrimination in the Health System. The revelation that the political activity on FM was lacking as far as discrimination against women was denounced in said debate: ‘in regard to the diagnosis and treatment in general, especially where diseases germane to women are concerned, such as fibromyalgia, breast and womb cancer, and osteoporosis’. A written European Declaration on FM was presented in 2008, which incited the development of a common strategy on FM which member states could use for diagnosis and treatment of the disease. So far, this has not been the case, nor has the disease been included in the EU official pathology index, which gave rise to a denouncement in 2010 of patients’ difficulty in obtaining an official diagnosis.

FM appeared for the first time in a Spanish parliamentary debate on gender discrimination in the healthcare system in 2005, when the shortcomings with regard to gender discrimination and the lack of attention received by women by the healthcare system were denounced:

there is discrimination as far as diagnosis and treatment in general is concerned, above all when the disease in question primarily affects the female population, such as fibromyalgia’. (Teresa Riera Madurell)

Another member of parliament denounced the invisibility of predominantly female diseases (FM and multiple chemical sensitivity) in 2005 and 2008 owing to their difficult diagnosis and the lack of resources assigned to research projects and treatment, the recognition of unfit for work status, and the necessity to launch an enquiry into said anomalies:

90% of people affected by these diseases are deemed to be women /…/ They are both gendered diseases, of challenging diagnoses, with minimal resources assigned to research and subsequent treatment, and which normally cause problems as far as the ability to work is concerned due to the lack of recognition of handicaps caused to the sufferer. The causes of these diseases need to be discovered, more effective treatments need to be developed, and the results need to be contrasted, along with a programme of social awareness and further information on their social, legal and workplace repercussions. (Kathy Sinnott, 2009)

In 2009, the fact that certain diseases affecting mainly women and which required further research into their causes or risk factors were on the increase was drawn attention to: ‘we should now dig deeper, and carry out thorough research on these diseases which are affecting women’.

### Part II: from the health professionals’ perspective

The main findings of the analysis of the interviews with professionals are: 1) dealing with FM is frustrating for healthcare professionals owing to the lack of effective treatment to offer to patients in order to relieve their pain and 2) healthcare professionals construct FM as being a women’s disease and assign negative stereotypes to patients suffering from it.

#### Clinical aspects of the management of FM: diagnostic approach, therapeutic approach, and the doctor–patient relationship

Without a sound diagnosis, we are dealing with the benefit of the doubt. Is it or isn’t it? (Male Occupational Physician)

The management of FM poses a challenge to health professionals, as no singular treatment exists for the disease, or one that was found to be effective. Furthermore, current treatment does not cure. Dealing with FM cases is challenging for professionals, due to the existing uncertainty surrounding the disease, especially regarding its aetiology. This lack of knowledge makes diagnostic and therapeutic procedures difficult, which in turn adds to the tension in the doctor–patient relationship. Professionals attribute the difficulty in being able to carry out a correct diagnosis to the lack of objective proof. The professionals feel largely to be of little use to such patients, as they are unable to offer them a fast and effective solution, and they in turn feel hopeless. The limitations as far as resources are concerned, for example the lack of time available in the surgery, have emerged along with other such difficulties when dealing satisfactorily with people affected. Professionals feel, on a regular basis, unable to help these patients, which adds to their frustration and clouds their relationship with their patients. Doubts about the real existence of the disease or simulation between patients are also highlighted.

#### Health professionals’ attitudes towards FM and its patients: the problematic women

‘You talk to rheumatologists and they say: oooh, how can you stand those women? (Female psychologist)

some people prefer not to use this diagnosis, because it will stigmatise the patient. (Male Rheumatologist)

Health professionals are aware of the lack of recognition existing on a social level, and, above all, in the health professional collective. They admit that prejudice is present when the subject of FM and its patients is brought up, and the fact that health professionals feel rejection towards such patients, especially women, is a common occurrence.

Professionals describe women patients with characteristics fitting well with the gendered stereotype of women weak, complaining, and overreacting; as well as conflictive patients, with a reivindicative sick role identity. They identify men as not seeking health care for these health problems, which reflect the traditional rejection of showing weakness assigned to the masculine identity.

Furthermore, the results suggest the existence of a gender-biased patient healthcare attention. In spite of the fact that the majority of these patients are women, a few men are also affected by the disease. The lack of recognition for the disease, together with the fact that said disease is socially constructed as a woman’s problem, could result in 1) a lack of effort in diagnosis, which in turn generates a possible over or under diagnosis, and 2) less concessions in applications for unfitness for work and disability allowances. It seems to be more difficult to obtain for women due to gendered stereotypes about their traditionally less prestigious and less exhausting jobs, and about the greater severity of the symptoms perceived in men, underestimating the claims of women.

#### Ideal types of health professionals attending FM patients

**Fig. 2:**
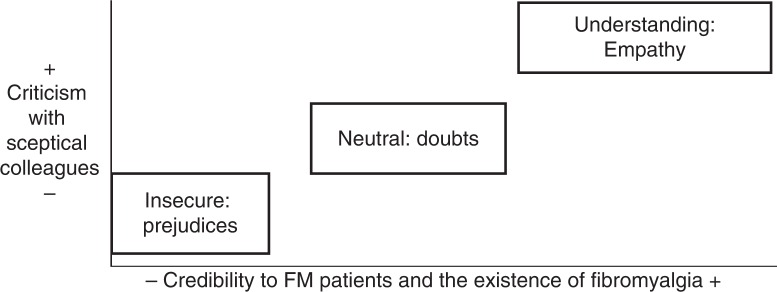
Ideal types of healthcare professionals and their attitudes towards fibromyalgia patients identified.

Ideal types of professionals and their attitudes towards FM and its patients emerged from the combination of the credibility given to the disease and the people affected by it, and the criticism or justification of attitudes held by their professional colleagues to FM (Fig. 2). One participant could contribute data to more than one ideal type, abstract definitions which only reflect prototypical descriptions of professional profiles. Health professionals interviewed ranged from those ones who think that patients are really suffering or just pretending, which is also common related with the stereotypical idea of women’s unnecessary complaints. Those health professionals who are more empathetic with patients’ suffering and who recognise the severity of the disease are also the ones who tend to criticise those health professionals who do not believe in the existence of FM as a real disease. Furthermore, the attitudes of healthcare professionals towards their patients have an influence on the quality of caregiving.

### Part III: from the patients’ perspective

FM has a negative impact on the lives of the people affected by it in three spheres – their working lives, social lives, and private lives. Patients feel that the passivity derived from fatigue caused by FM limits their working lives, the relationship with their family, and their intimate relationships. The results show that FM sufferers 1) feel misunderstood by healthcare staff and their support network, as their disease has little social recognition, 2) they have to struggle to maintain their own identity, and 3) their lives remain tainted by a gendered division of labour, both inside and outside the home.

#### Patients’ perceptions of the clinical management of FM

I feel terrible, but why do I have to go around with huge bags under my eyes and my hair a mess so that they see that I’m not well? (Woman with FM)

Patients state that the obtainment of a diagnosis was a challenge, after having waited for years or even having been given an incorrect diagnosis. Furthermore, some patients are reluctant to seeking help from mental health services, rejecting the idea that psychological factors may be involved in the aetiology of this disease. The doctor–patient relationship is described by patients as unsatisfactory mainly due to the lack of empathy and understanding recognised by their healthcare providers. Patients seek a good level of communication and understanding when dealing with professionals, they like to feel supported in their search for effective solutions (by way of tests and endeavour) and to find a human interest in the follow-up procedures that are used on them, which in itself is therapeutic as far as the patients are concerned.

#### Ideal types of FM patients

**Fig. 3:**
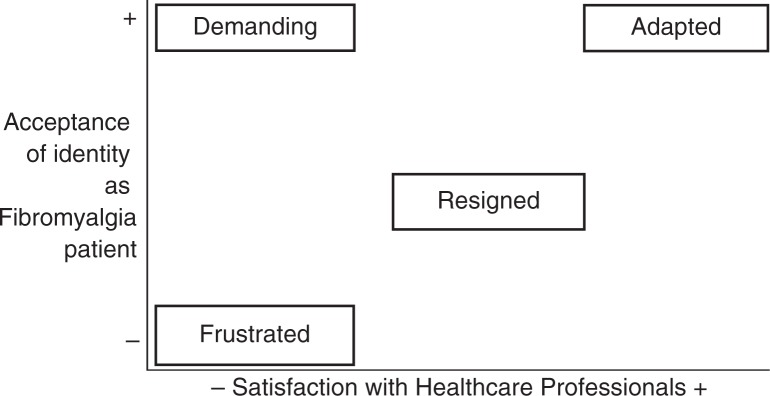
Ideal types of patients with fibromyalgia identified.

The results of this doctoral thesis have also enabled the establishment of ideal patient profiles (Fig. 3). These prototypes are defined by the convergence of two factors: the identity ascribed to those affected as FM patients, that is to say, the assumption and acceptance of suffering from the disease, in what measure they identify with the rest of FM patients and the adaptation to the consequences of FM in their lives; and, on contrary, satisfaction with the healthcare system – which should be more of an optimum doctor–patient relationship based on empathy, understanding, patience, and orientation, rather than successful therapy, as results have shown.

It is during this phase of undergoing diagnosis when the person affected starts to feel that their identity is under threat. The ideal type of frustrated patient coincides with the first step of the adaptation process. Here, denial is the defence mechanism used; he/she refuses to see himself/herself as a sick person, does not accept the assigned diagnosis, rejects the idea of belonging to the group of FM sufferers, and feels that the health care they have received has been lacking.

The second stage the patient goes through is that of resignation: acceptance of diagnosis, feeling hopeless, giving up seeking a solution, and feeling let down by the health system. The expectations of an ideal patient would be one where defeat would not take place at this stage of the process, but – in accordance with the prototype of the assertive patient – would seek solutions and fight for ways to improve their quality of life even though the system does not offer them social recognition, which is something FM sufferers need and miss. The ideal assertive patient would not blindly accept an ineffective medication prescribed by a doctor, or if a doctor lacked the necessary skills to assess him/her correctly. He/she would fight either on an individual or collective basis, joining forces with patients’ rights groups.

Finally, the ideal adapted patient would have achieved a balance between his/her identity taken on as a patient with a chronic, incurable, and limiting disease such as FM, would have learned to live with the symptoms, and the results from the search for therapeutic solutions from either one or several professionals who provide a satisfactory service, or at least one that does not create problems: efficient in terms of fulfilling the expectations of feeling accompanied in the whole process, being taken seriously and understood.

This final stage of a hypothetical development undergone by a patient would be provided by a co-ordination between multidisciplinary teams or between several professionals to adapt a personalised programme of therapy to each case and secure an effective follow-up of the patient’s progress sustaining effective communication among themselves.

#### Patients’ perceptions of the impact on their daily lives

because of my illness, I couldn’t earn enough money, which has affected me personally. My wife had to go out to work. (Man with FM).

As long as I can clean, here I stand (Woman with FM)

Those affected by the disease, above all, wish to maintain their identity as people who are not sick. This is carried through by trying to keep up their daily routine. To be able to carry on working in a job adapted to their needs is of benefit to patients, depending on the degree of handicap involved, this being the case for both the women and men who were interviewed. In turn, caring for family members and doing housework make women’s symptoms worse; however, not to carry out these duties brings on feelings of guilt only in the case of the women. This was not the case for the men who were interviewed, however. Not being able to fulfil the gendered expectative of being the breadwinner of the family had worse emotional consequences in the case of men than the women interviewed. Men with FM feel guilty that their wives have had to go out to work.

To women, the assumption of the traditional female role is revealed as an adaptation mechanism to the disease. This contributes to their self-esteem and makes them feel useful, as opposed to feeling a burden to the family. However, women continue to take on the exclusive role as far as childcare is concerned, and men are absent as far as domestic responsibility is concerned, such as housework.

Roles involving the division of labour assigned to patients according to their sex were found to follow a rigid pattern. It is apparent that the women affected by FM wish to fulfil their household duties and act as a carer to their families. The burden in carrying out both roles (public – work outside the home, and private – household duties) defines their chosen path. In the domestic sphere, the burden women have to bear due to a marked definition in gender roles can aggravate the suffering and symptoms ascribed to FM.

The responsibility as far as childcare is concerned has completely different implications for the men and women taking part in the interview. The main concerns that the men had were not being able to take part in leisure activities outside the home, not being able to support the family financially and, thus, secure a prosperous and self-sufficient future for their children.

## Discussion

The study comprising this doctoral thesis shows how FM is considered to be one of the ‘invisible female diseases’, in which health policies do not afford them the justice they deserve, the patient receive it neither in the field of health care nor in the private or public sphere of the sufferer. The same thing applies to other pathologies affecting women such as endometriosis or those that are largely diagnosed, as in anxiety and depression, which have been assigned to women in a historical sense due to a supposed greater mental frailty and vulnerability as far as the female gender is concerned (30–33). The classification of diseases and syndromes is in accordance with biomedical paradigms based on physiopathology and an objective position (7) and, consequently, the biologistic approach is unable to provide answers when changes found in signs and symptoms are not brought about by organic causes (34). As a result, diseases such as FM remain outside the biomedical classical ‘system’, which aims to identify the organic cause of disease and treat symptoms with drugs (35). This may explain why health policies on an international level have not yet taken the emergence of this recently diagnosed disease into consideration. The invisibility of FM from health plans is especially worrying because government plans, specifically health plans, are the result of the ‘political construction’ of the problems, which have succeeded in being established in the political agenda (36). In the case of FM, visibility has not been successful, in spite of FM being recognised as a disease in the WHO in 1992 and taking into account its high prevalence. To omit health problems prevalent in women, such as FM, obstructs offering a health service on an equal basis and gives rise to a possible imbalance towards the needs of women (11, 37). The increase in the number of FM units on a national basis, where professionals involved in the assistance of such patients are coordinated, has been identified as a possible solution in improving the management of the affected people. In fact, this was one of the demands included in Spanish parliamentary initiatives. In turn, the increase in parliamentary interest is having a positive impact, as this brings the general public’s attention to the knowledge and social awareness on this particular health issue via the media, as is the case with other health issues (38, 39).

According to previous studies, it is known that limited social recognition of the disease is coupled with the problems inherent in suffering from FM, which leads to isolation and a decrease in interpersonal relationships (40, 41). People affected are obliged to undergo an adaptation process, in which family members must also take an active involvement, in line with the recommendations of previous studies (42, 43). In relation to that, Fig. 3 makes sense when it is placed in relation to the explanations found in the literature on the adaptation process, which FM sufferers must face (44). Said process can be set out by a division of the following phases: explaining previous personal experience to FM; being given a diagnosis and accepting it; negative feelings towards oneself upon the manifestation of suffering; feeling trapped in ones’ own body; adaptation to FM; and continuing to fight for a better quality of life (45).

This study shows that there are professionals who have doubts on whether FM actually exists as a real disease in itself. Doubts as to the existence of the disease or the simulation among patients, which condition medical and administrative decisions involved in the recognition of the existence of any handicap, have been already documented (46, 47). This study went further and claimed that the lack of understanding inherent in employers, colleagues, and healthcare staff may be partially explained by the existing uncertainty, as to its aethiopathogenia, together with the fact that the majority of patients are women. According to the literature, the stereotypes and the prejudice of healthcare staff taints their behaviour when interacting with patients, along with making clinical decisions in connection with diagnosis and prescribed therapy (48, 49). In fact, the results show a possible gender bias in the diagnostic and sick leave permissions given: while it is more common diagnosing FM in women, more men obtain the official recognition of the severity of their symptoms through a sick leave concession. In this thesis, I theorised that diagnosis could prove difficult for male patients due to the fact that, maybe, on an unconscious level, the health professionals themselves foster the idea that this is a socially accepted women’s disease. Besides, generally, men look for less medical attention because the unequal gender socialisation prevents them to show weakness (50).

Applying gender theory, which outlines and denounces gender equality in society, has been useful to explain how gender stereotypes have an influence on the social construction of the problem being one of a women’s health issue and the lack of recognition it receives. In the case of women, the lack of inherent credibility is made worse by the prejudice that traditionally falls on them by exaggerating the scale of their problems. This finding is related to the disease being associated with complaining patients and the social discreditation of the disease, which converges with the feminine identity assigned to it. Therefore, the severity of the symptoms and the complaints made by those suffering from FM could be underrated (51). These results back the idea that diseases mainly attributed to women have low prestige and recognition among healthcare professionals (5, 41, 52).

Since gender bias in FM is an important issue that is overlooked in the FM management guidelines, there is a need to develop specific intervention programmes with strategies that address negative attitudes and stereotyping in health service providers. Health professionals need to be made aware of emergent diseases affecting mainly women, as well as improving their communication skills with patients with painful chronic diseases, since an empathetic relationship between them is in itself therapeutic. Health practitioners should also take measures to better understand their patients’ feelings so as to be more sensitive to their needs (53). Nevertheless, the healthcare system does not always provide training for clinicians on appropriate attitudes and sensitivity towards patients with emerging diseases (53, 54). Advances in dissolving gender stereotypes could contribute to more effective management of the challenges of patients with FM (55). For example, training for undergraduate and postgraduate medical staff would improve their knowledge and competencies regarding diagnosing FM and providing support to patients; it also will help health professionals to be sensitive to the needs of both women and men and to increase awareness of the existence of stereotypes about women suffering from FM (56, 57).

The social awareness on the severity of FM and the removing of gender stereotypes necessitates the participation of a variety of agents, among whom those who promote policies play a primordial role. The development of specific strategies and measures in the health sphere centring on FM primarily needs to be incorporated in the health agenda, after having been previously incorporated in the political agenda. That is to say that parliamentary initiatives are able to generate new policies, such as laws or guidelines for action, both of a financial and healthcare nature, promote and develop new techniques to detect an early diagnosis, improve healthcare personnel training, and implement campaigns, providing information and social awareness on the disease.

Among the limitations of this study, the small sample of men affected by the disease brings to the necessity to continue the research exploring the consequences of suffering from an unrecognised and feminised disease such as FM on men and to continue this line of research from a gendered perspective. Although there could have been differences and similarities between the different types of health professionals, for example, according to professional profile or gender, we were not able to identify such differences in this study, and attention was centred on a general discourse without said differentiation. Future work should focus on factors that lead to gender differences in FM diagnosis and gender-specific treatment strategies.

## Conclusions

The disease’s lack of recognition is still common in the policies, the professional and clinical fields, and the affected people’s social circle. Many factors have an influence on how this disease is socially constructed. FM is looked upon as a disease of minor importance, as the majority of patients are women – and as a consequence, due to the pervasion of certain negative stereotypes as far as women and their health are concerned (tendency towards subordination, dramatisation, and mental and physical weakness). FM can be considered as one of the ‘invisible women’s diseases’, not being considered as important as they should be as they belong to a group of pathologies, which are predominant in females. This, in turn, has an impact on 1) how health professionals approach such patients in their surgeries and 2) how people affected by FM live with the consequences of the disease on a daily basis.

## Paper context

This article seeks to further integrate the gender perspective with the social construction of fibromyalgia (FM) in the policy, social, and private fields. Although FM is majorly reported in women, there is a dearth of research studies on fibromyalgia that address the gender perspective, especially since ‘gender’ influences women’s daily lives to a great extent. As the socialisation process constructs feminity as culturally and socially opposite to masculinity, health problems are socially constructed by the social actors involved. To my knowledge, this is the first such study on FM carried out in Spain.
